# Genome-wide atlas of rust resistance loci in wheat

**DOI:** 10.1007/s00122-024-04689-8

**Published:** 2024-07-09

**Authors:** Jingyang Tong, Cong Zhao, Dan Liu, Dilani T. Jambuthenne, Mengjing Sun, Eric Dinglasan, Sambasivam K. Periyannan, Lee T. Hickey, Ben J. Hayes

**Affiliations:** 1 Queensland Alliance for Agriculture and Food Innovation, The University of Queensland, St Lucia, QLD 4072 Australia; 2 National Wheat Improvement Centre, Institute of Crop Sciences, Chinese Academy of Agricultural Sciences, Beijing, 100081 China; 3 School of Agriculture and Environmental Science and Centre for Crop Health, University of Southern Queensland, Toowoomba, QLD 4350 Australia

## Abstract

Rust diseases, including leaf rust, stripe/yellow rust, and stem rust, significantly impact wheat (*Triticum aestivum* L.) yields, causing substantial economic losses every year. Breeding and deployment of cultivars with genetic resistance is the most effective and sustainable approach to control these diseases. The genetic toolkit for wheat breeders to select for rust resistance has rapidly expanded with a multitude of genetic loci identified using the latest advances in genomics, mapping and cloning strategies. The goal of this review was to establish a wheat genome atlas that provides a comprehensive summary of reported loci associated with rust resistance. Our atlas provides a summary of mapped quantitative trait loci (QTL) and characterised genes for the three rusts from 170 publications over the past two decades. A total of 920 QTL or resistance genes were positioned across the 21 chromosomes of wheat based on the latest wheat reference genome (IWGSC RefSeq v2.1). Interestingly, 26 genomic regions contained multiple rust loci suggesting they could have pleiotropic effects on two or more rust diseases. We discuss a range of strategies to exploit this wealth of genetic information to efficiently utilise sources of resistance, including genomic information to stack desirable and multiple QTL to develop wheat cultivars with enhanced resistance to rust disease.

**Supplementary Information:**

The online version contains supplementary material available at 10.1007/s00122-024-04689-8.

## Introduction

Wheat (*Triticum aestivum* L.) is the second most cultivated staple crop, providing about one-fifth of the calories and proteins for the global human population (FAOSTAT, 2019; http://www.fao.org/faostat). The sudden interruption of this supply could cause serious famine and social disturbance (Anderson 2012; Bentley 2022). To meet future demand, wheat production must increase by 60% by 2050 (https://www.wheatinitiative.org). This highlights the importance of wheat breeding to improve crop productivity and resilience in the face of biotic and abiotic stresses. Globally, pests and diseases cause an average annual reduction to wheat yield of 21.5% (Savary et al. 2019). In particular, wheat rust diseases caused by biotrophic fungi *Puccinia* sp. are responsible for major yield and economic losses. Leaf rust is prevalent in almost all wheat-producing areas and has been reported to cause large economic losses in Australia, New Zealand, and the USA (Huerta-Espino et al. 2011). Stripe rust occurs in over 60 countries, almost on every continent, with major epidemics often reported in Ethiopia, the USA, Australia, and China (Wellings 2011; Savary et al. 2019). New and highly aggressive rust pathotypes have emerged in recent years, posing a significant threat to food security. For example, the *PstS4* lineage races caused significant outbreaks of stripe rust on Triticale in Scandinavia in 2009, and subsequently, the *PstS7* (Warrior), *PstS8* (Kranich), and *PstS10* [Warrior(−)] lineages have largely dominated in Europe (Bouvet et al. 2022). In the US, different leaf rust pathotypes have been described recently, with TBBGS, MNPSD, and BBBQB being the most prevalent (Kolmer et al. 2019). Serious production damage was caused by stem rust in Africa and the Middle East due to the emergence and spread of the highly virulent TTKSK (Ug99) race, which has now reached South Asia (Singh et al. 2011; Patpour et al. 2024). However, other lineages not related to Ug99 can also cause significant crop loses, such as TRTTF and TKTTF (Olivera et al. 2015). Based on available estimates, leaf, stripe, and stem rust diseases can result in wheat yield losses up to ~ 50% (Huerta-Espino et al. 2011), ~ 70% (Chen 2020) and ~ 100% (Soko et al. 2018), respectively, during epidemic outbreaks under favourable conditions.

The leaf, stripe, and stem rust diseases of wheat are caused by *P. triticina* (*Pt*), *P. striiformis* f. sp. *tritici* (*Pst*), and *P. graminis* f. sp. *tritici* (*Pgt*), respectively. Incidence of the three rusts depends on climatic conditions and the genetic background of the host for resistance. Generally, cool and wet weather (12–20 °C) provides *Pst* with optimal conditions, while hot and dry conditions significantly impair disease development and spread (Chen 1995). In contrast, the optimal temperature for infection of *Pgt* is much warmer (15–30 °C) (Singh et al. 2011). Warm and intermediate temperature conditions (15–25 °C) are optimal for *Pt* infection and spore production on cereals (Singh and Rajaram 1992; Bolton et al. 2008). This strong temperature dependence often determines the timing of the different rust diseases in wheat crops. For example, in Australia, stripe rust predominately occurs in the early developmental stage of wheat, which is during winter with cooler temperatures, followed by leaf rust occurrence in spring as temperature starts raising, and finally, the incidence of stem rust at the crop maturity stage during the summer. Subsequently, the pathogen evolves virulence through spontaneous mutation and somatic hybridization (Chaves et al. 2013; Li et al. 2019). The pathogen also tends to evolve virulence through infection on alternate hosts where they complete the sexual life cycle and undergo recombination (Li et al. 2023; Sperschneider et al. 2023).

Of the main approaches to control rust epidemics (viz*.* fungicides, quarantine, and resistance breeding), the deployment of host plant-mediated genetic resistance is widely preferred due to relatively low cost and minimal environmental impact (Jørgensen et al. 2017). Genetic resistance to rust diseases can be divided broadly into all-stage resistance (ASR) and adult-plant resistance (APR), according to the plant growth stage when resistance is phenotypically expressed (although a few resistance genes do not fall into both categories (Neu et al. 2002)). ASR is effective across all growth stages from seedling emergence, while APR typically provides intermediate levels of resistance that is more pronounced in its phenotypic expression at adult growth stages. ASR is typically underpinned by a major gene acting in a gene-for-gene manner, where the recognition of a specific effector or avirulence (Avr) molecule secreted from a selected strain of the pathogen during infection is essential for an incompatible reaction to occur. Hence, ASR genes are also referred to as race-specific resistance genes (Sánchez-Martín and Keller 2021). ASR genes generally minimise the reproduction ability of the pathogen through hypersensitive reactions, in which plant host cells undergo programmed cell death to stop the spread of the invading fungal pathogen (Lu and Tsuda 2021). However, this can apply strong selection pressure for mutations in the pathogen population that ultimately lead to the increased frequency of virulent pathotypes. APR is often underpinned by multiple minor genes, which tend to confer non-race-specific resistance (Park and McIntosh 1994; Sørensen et al. 2014). However, this is not always the case, for example, APR genes expressed at early growth stages (e.g. fourth leaf stage), such as *Lr49* and *Lr22a*, are known to confer race-specific resistance to leaf rust (Norman et al. 2023). Most APR genes decrease disease severity by increasing the latency period and/or inhibiting the degree of pathogen sporulation, as well as reducing infection frequency and uredinia size and, in most cases, do not arrest the growth of the pathogen completely. As a result, individual resistance alleles exert low selection pressure on the pathogen population to evolve virulence over these genes (Ellis et al. 2014). For the above reasons, APR genes have also been described in the literature as horizontal, partial, slow-rusting, or durable resistance genes.

Rust pathologists and wheat breeders have sourced resistance (*R*) genes from a wide array of sources, including primary (cultivars, landraces, and wild progenitors of common wheat with homologous genome), secondary (domesticated *Triticum* and *Aegilops* relatives with at least one homologous genome of common wheat), and tertiary (distantly related species without homologous genome of common wheat) gene pools (Zhou et al. 2020). The first quantitative trait loci (QTL) mapping in wheat was reported in 2000, wherein a locus for stripe rust resistance was positioned using first-generation markers (restriction fragment length polymorphism; RFLP), which were developed based on the restriction site differences between genomes (Börner et al. 2000). Later studies used PCR amplification-based markers such as random amplified polymorphic DNA (RAPD), amplified fragment length polymorphism (AFLP), and simple sequence repeats (SSRs). Among these marker types, SSR markers, which tag DNA nucleotide repeat differences, were widely used due to high levels of polymorphism, codominance, and ease of use (Röder et al. 1998). However, the advent of next generation sequencing (NGS) technology led to the high-throughput and cost-effective array- and sequencing-based genotyping systems, which enabled faster, more accurate, and cost-effective mapping using single nucleotide polymorphism (SNP) markers, distributed abundantly in any given genome. Today, SNP-based arrays, such as the 90K SNP chip, Diversity Array Technology Sequencing (DArTSeq), and genotyping by sequencing (GBS) platforms are the most commonly used tools for QTL discovery studies in wheat (Rasheed et al. 2017). In parallel, efforts to refine the wheat genome assembly have greatly accelerated the genetic dissection of traits, including mapping, marker development, and gene cloning. For instance, the fully annotated reference genome of common wheat Chinese Spring (CS), RefSeq v1.0, was released in 2018, and the pangenomes for 10 widely used wheat cultivars with reference-quality pseudomolecule assemblies were made available in 2020 (IWGSC, 2018; Walkowiak et al. 2020). These genomic resources have already assisted the discovery of new rust *R* genes and allelic diversity (Mackenzie et al. 2023; Zhu et al. 2023).

Even prior to the release of the wheat genome assembly, a multitude of QTL related to rust resistance were identified using linkage mapping or association mapping approaches, as described above and evident from hundreds of publications. However, the substantial number of QTL reported using diverse donor sources, population structures, and types of molecular markers made it difficult for researchers to determine whether a mapped locus was novel or not, and as a result, contributed to a high degree of redundancy in the QTL reported. To effectively leverage historic studies and prepare for future studies, the genomic intervals containing rust resistance loci should be collated. The recently updated CS reference genome (RefSeq v2.1; Zhu et al. 2021) now enables the precise integration of genetic and physical maps. Therefore, the physical positions and breeding values of reported QTL can be collated and more accurately compared through the effective use of flanking marker sequences. This would consolidate results from previous studies and help identify high-value genomic regions for resistance breeding and gene-cloning efforts.

In the first section of this review, we summarise previously mapped QTL and cloned *R* genes and position them onto the 21 wheat chromosomes to create a ‘Genome Atlas’ for rust resistance loci to guide future research and breeding. Physical locations using the RefSeq v2.1 assembly were identified for each QTL using midpoints of the genetic intervals predicted from the flanking or the closest linked marker positions. Presence of more than one QTL on the same chromosome was indicated if the distance between QTL was larger than 10 Mb, while QTL-rich clusters (QRCs) were classified if the distance was < 10 Mb, a conservative estimate that reflects the precision of historical QTL mapping studies (Cao et al. 2020; Tong et al. 2020; Xu et al. 2023). In the second section, we outline strategies for rust resistance breeding that leverage the resultant genetic resources. Our review summarises the genetic architecture of rust resistance in wheat, which is anticipated to accelerate the effective utilisation of resistance from diverse resources, support rapid selection based on genomic information, and stacking of desirable QTL for additive and durable resistance.

## Collating and comparing QTL and cloned *R* genes underlying wheat rust resistance

### Positioning previously reported QTL detected through linkage mapping and classification of QRCs for rust resistance

**Fig. 1:**
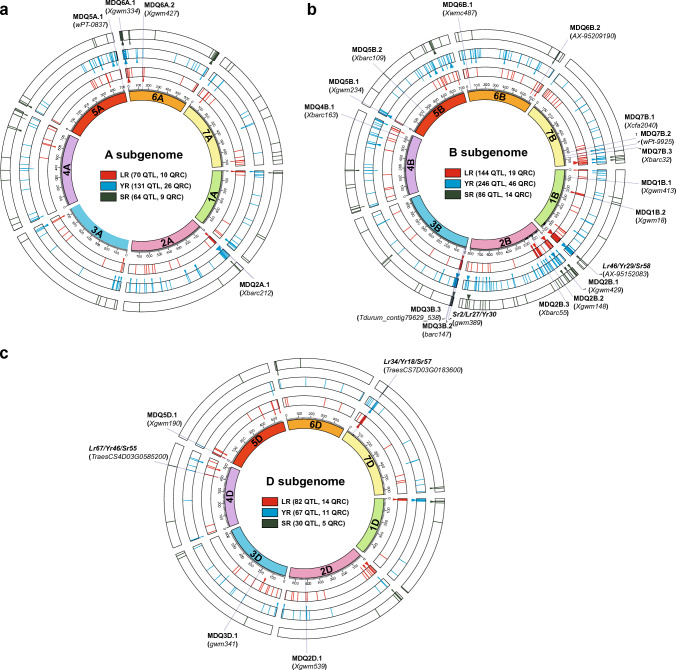
Landscape of genetic architecture underlying leaf rust (LR), stripe/yellow rust (YR), and stem rust (SR) on wheat genome. **a** A sub-genome; **b** B sub-genome; **c** D sub-genome. The innermost ring indicates wheat chromosomes. The outward rings represent LR, YR and SR in sequence. Coloured triangles point to QTL-rich clusters (QRC) of each disease. Potential multi-disease QTL (MDQ) are denoted by transparent purple bars. The names of each MDQ are displayed, including associated marker in parentheses

QTL analysis by linkage mapping has contributed greatly to identification of genetic loci underlying the target traits based on the frequency of recombination determined by the genetic distance between the two linked loci within a bi-parental family segregation. The method accelerates the tracing of the source of resistance and allows application of diverse genetic loci in resistance breeding. In this review, 920 previously mapped QTL for the three wheat rusts were summarised from 170 publications (48, 90, 32 publications on leaf, stripe, and stem rust, respectively) (Table S1; Table S2). The information collected and compiled from each study includes: (1) year of the study; (2) parental lines used for crosses, type and size of mapping population; (3) the type of environment and the number of QTL collected; (4) QTL name(s) and donor source of resistance allele(s); (5) flanking or closest linked markers of each QTL; and (6) logarithm of the odds (LOD) score and phenotypic variation explained (PVE) or *R*^2^ value of each QTL. All of the 920 collected QTL were mapped onto the latest wheat reference genome IWGSC RefSeq v2.1 as following: (1) the sequences of the closest linked markers or two flanking markers on both sides of QTL confidence interval were retrieved from publications or databases including 90 K (Wang et al. 2014), 660 K (Cui et al. 2017), CerealsDB (https://www.cerealsdb.uk.net/), GrainGenes (https://wheat.pw.usda.gov/GG3/), DArT (https://www.diversityarrays.com), and URGI (https://wheat-urgi.versailles.inra.fr/); (2) physical positions of above marker sequences were obtained from RefSeq v2.1 assembly using local BLASTN; (3) physical locations of each QTL were identified using midpoints of the confidence intervals defined from both sides of flanking or the closest linked marker positions. Formally named and cloned genes were also incorporated in the list (Table S2). Figure 1 displays the genome-wide genetic landscape for resistance against the three rusts, and Fig. S1 labels formally designated and cloned resistance genes. For leaf rust, 296 genetic loci were summarised and integrated onto the wheat chromosomes based on their physical locations (Table S2). Among them, at least 246 previously mapped loci, as well as 50 formally designated QTL and cloned *R* genes, were included. When genome-wide distributions of the resistance loci were shown in Fig. 1, different distribution patterns were observed at the chromosome level, although all 21 chromosomes contained QTL for leaf rust resistance. Among the three sub-genomes, the B genome harboured 144 QTL, which was far greater than the A (70) and D (82) sub-genomes. Interestingly, the homeologous group 2 chromosomes contained the most QTL (66), followed by group 1 (58) and 7 (52), while the group 4 chromosomes had the lowest number with 26 QTL. In terms of individual chromosomes, 1B, 2B, 7B, 2D, and 6B harboured more than 15 QTL, whereas 6D only had four for leaf rust resistance, representing the lowest number. The greatest number was mapped to chromosome 1B, where 38 QTL were identified from different studies, indicating the importance of this chromosome for leaf rust resistance.

We summarised 406 previously mapped loci for stripe rust resistance from 90 publications (Table S1; Table S2). Coupled with 39 formally named or cloned genes, these genetic loci were all positioned in Fig. 1 and Fig. S1 to visually illustrate the genetic basis of stripe rust resistance. Similar to leaf rust, a rich source of stripe rust resistance loci was evident in the B sub-genome, where 246 QTL were located. This result may be partly due to the larger number of markers available on the B sub-genome compared to the A and D genomes in most wheat genetic studies. The D sub-genome contained the least stripe rust resistance QTL (67), in contrast to the result for leaf rust resistance. Group 2 chromosomes held the maximum number (103) of stripe rust resistance QTL, whereas group 4 harboured the lowest number (48), which was similar to leaf rust resistance, implying multiple genes conferring rust resistance were on group 2 chromosomes but the contribution from group 4 was relatively minor. At the individual chromosome level, a large number of QTL for stripe rust were reported on 2B (56), followed by 1B (43), 2A (36), 3B (34), and 5B (32). Only three QTL were located on 5D.

For stem rust, we summarised 180 loci distributed across all 21 wheat chromosomes (Table S2; Fig. 1). Group 2 chromosomes carried 38 QTL for stem rust resistance, which was the highest among the seven chromosome groups, whereas group 5 had the least (17). At least ten QTL have been reported on chromosomes 2B, 6A, 3B, 4A, 5B, and 4B, but only one QTL was identified on 5D based on our literature search. Overall, there appeared to be a somewhat unbalanced distribution of rust resistance loci across the chromosomes of hexaploid wheat.

If the same or closely linked QTL are identified in different studies, it builds confidence in the genomic region for resistance breeding. For instance, if QTL are detected in different genetic backgrounds and/or environments, they could potentially confer relatively long-term, broad-spectrum or large-effect resistance. To identify such regions, we defined a QRC as genomic regions containing at least two QTL from different studies that were positioned within 10 Mb. This criterion is the same applied in several recent publications identifying QRC for plant height (Xu et al. 2023), yield components (Cao et al. 2020), and micronutrients (Tong et al. 2020) in wheat. Such a QRC is likely to indicate there is at least one reliable QTL underpinning the given trait. It is worth pointing out that it is possible for QRCs to contain multiple tightly linked QTL from different studies—this may be resolved in future through more diverse and larger-scale mapping studies. In total, we identified 154 QRCs for rust resistance in the wheat genome (Table S3; Fig. 1). Consistent with the number of QTL we collected, a highest number of QRCs were identified for resistance to stripe rust (83), compared to leaf rust (43), and stem rust (28). Regarding chromosomal distribution, the A, B, and D sub-genomes contained 45, 79, and 30 QRCs, respectively. Hence, the B sub-genome appears to contribute more genetic variation for rust resistance than A and D. All chromosomes contained QRCs for leaf and stripe rust resistance, with the exception of chromosome 6D, and no QRCs for stem rust resistance were identified on chromosomes 3A, 3D, 4D, 5D, and 7A. Notably, 15 QRCs were identified for stripe rust resistance covering almost all regions on chromosome 2B, highlighting the chromosome as a ‘hot spot’ for multiple resistance loci for stripe rust. In the QRC intervals, there were several formally named or cloned *R* genes (Table S3). Given they contain information from multiple studies, QRCs offer more confidence for further analyses, such as fine mapping and isolation of the causal gene(s) responsible for the resistance.

### Comparison of QRCs with marker-trait associations detected in genome-wide association studies (GWAS)

GWAS is an effective and widely used approach to detect marker-trait associations based on linkage disequilibrium (LD) and historical recombination of chromosome segments present in a population (Hamblin et al. 2011). The method can complement QTL linkage analysis, but many more markers are required in high-resolution GWAS mapping due to abundant recombination events and the limited extent of LD versus linkage in populations. GWAS has become the method of choice with the rapid development of marker and sequencing technologies, particularly the availability of high-density SNP arrays and re-sequencing techniques (Liu et al. 2023). In our review, we collected results from 15 GWAS studies (seven, nine and four related to leaf, stripe, and stem rust resistance, respectively) conducted over the past five years (Table S4), where various wheat diversity panels with different population sizes were phenotyped and studied across 15 countries. Most of them were performed with SNP arrays, and three used the DArT genotyping platform. In total, 317 marker-trait associations for rust resistance were summarised in Table S5 with available physical locations and detailed information. The reported marker-trait associations were compared with the QRCs (defined above) based on their physical positions using the IWGSC RefSeq v2.1 (Table S5). As anticipated, a large number of QRCs (41 out of 154) aligned well with at least one marker-trait association. However, not all QRCs identified by bi-parental population studies were also identified through GWAS. Both linkage mapping and GWAS approaches have their strengths and weaknesses for QTL discovery. For example, GWAS typically has low power to detect rare alleles present in diversity panels. Nevertheless, both methods are complementary and integrating results can help to shed new light on the genetic basis of wheat rust resistance.

### Characterised genes responsible for wheat rust resistance

The first wheat rust resistance genes (*Lr10* and *Lr21*) were isolated successfully in 2003 (Huang et al. 2003; Feuillet et al. 2003). Since then, the development of a high-quality wheat genome assembly coupled with the introduction of rapid resistance gene-isolation strategies (Periyannan 2017) has greatly accelerated *R* gene discovery. Since 2003, nine genes that confer race-specific resistance to leaf rust have been cloned (Table S6). This includes *Lr1*, *Lr9*, *Lr10*, *Lr13*/*Ne2*, *Lr14a*, *Lr21*, *Lr22a*, *Lr42*, and *Lr58*, of which four were sourced from common wheat, located on chromosome arms 1AS (*Lr10*), 2BS (*Lr13*/*Ne2*), 5DL (*Lr1*), and 7BL (*Lr14a*), respectively. *Lr1*, *Lr10*, *Lr13*/*Ne2*, *Lr21*, *Lr22a*, and *Lr42* are canonical *R* genes encoding nucleotide-binding domain leucine-rich repeat (NLR) immune receptors. The *NLR* gene *RGA2* at the *Lr10* locus is required for activating *Lr10*-mediated resistance, where *Lr10* serves as a decoy to interact with pathogen effectors, and *RGA2* activates the downstream immunity (Loutre et al. 2009). *Lr13* is an allele of *Ne2* causing hybrid necrosis and also induces high-temperature adult-plant (HTAP) resistance to leaf rust (Hewitt et al. 2021; Yan et al. 2021). *Lr42* shows broad-spectrum resistance to many leaf rust strains and has been widely introduced into International Maize and Wheat Improvement Center (CIMMYT) wheat breeding lines (Lin et al. 2022). *Lr9* and *Lr58* encode identical tandem kinase fusion proteins conferring broad-spectrum resistance to various *Pt* strains, suggesting they were the same genes introgressed independently from two different species, *Ae. umbellulata* and *Ae. triuncialis*, respectively (Wang et al. 2022b). *Lr14a* encodes an unusual ankyrin transmembrane protein containing twelve ankyrin (ANK) repeats and six transmembrane helices (TM) with similar structure and function to calcium cation channel (Kolodziej et al. 2021). The gene is transcriptionally induced by avirulent *Pt* races and could be involved in calcium fluxes. In contrast to other *ASR* genes, *Lr14a* confers “mesothetic” seedling resistance, in which hypersensitive flecks and rust sporulation occurs simultaneously on the same leaf (Kolodziej et al. 2021).

To date, nine genes conferring resistance to stripe rust have been cloned (Table S6). *Yr36* displays APR and race-nonspecific resistance, whereas the others are ASR and race-specific genes. *Yr10*, *Yr27*, and *Yr28* encode typical NLR proteins, while *Yr5a*/*Yr5b* and *Yr7* encode an unusual NLR protein with N-terminal zinc-finger BED (*Drosophila* protein Boundary Element-Associated Factor and DNA Replication-related Element binding factor) domain. Interestingly, the recently cloned *YrNAM* encodes a NLR with zinc-finger BED and a NAM (No Apical Meristem) domain (Ni et al. 2023). Both these domains are required for stripe rust resistance. New evidence has indicated that *YrNAM* is very likely *Yr10* (Dibley et al. 2024). *Yr27* is allelic to *Lr13*/*Ne2* with 97% similarity at the protein level, implying diverse resistance function of a single *R* gene family (Athiyannan et al. 2022a). *Yr28* from *Ae. tauschii* is an interesting *NLR* gene with duplicated 3’UTRs that leads to transcription of at least four transcripts whose coordinated functions remains essential for the resistance against stripe rust (Zhang et al. 2019a; Athiyannan et al. 2022b). *Yr15* belongs to the tandem kinase-pseudokinase (TKP) family, where the pseudokinase domain could act as a decoy to bind with pathogen effector and switch the activation of the kinase domain (Klymiuk et al. 2018). *YrU1*, derived from *T. urartu*, encodes an ANK-NLR-WRKY protein, where WRKY domain could recognise the effector, leading to the homodimerization of ANK and CC domains and the activation of immune response (Wang et al. 2020).

On the other hand, 15 genes conferring pathogen-specific resistance to stem rust have been successfully cloned using wheat-related species (Table S6). Traditional map-based cloning method was employed to isolate *Sr13*, *Sr21*, *Sr33*, *Sr35*, *Sr50*, and *Sr60* (*WKS2*). Using the *R* gene enrichment sequencing with mutational genomics (MutRenSeq) method, five stem rust resistance genes *Sr22*, *Sr26*, *Sr27*, *Sr45,* and *Sr61* were rapidly cloned (Luo et al. 2022; Steuernagel et al. 2016; Upadhyaya et al. 2021; Zhang et al. 2021). Association genetics combined with NLR enrichment (AgRenSeq) was used to isolate *Sr46* and *SrTA1662* (now *Sr66*) (Arora et al. 2019; Cavalet-Giorsa et al. 2023). Recently, *Sr62* was successfully isolated from *Ae. sharonensis* (AS-1644) using genome sequencing and a high-quality assembly (Yu et al. 2022). Notably, all the above genes belong to the conventional NLR family except two TKP genes *Sr60* and *Sr62*. Notably, *Sr13* and *Sr21* confer stronger resistance to *Pgt* races at higher temperatures (24℃) by enhancing expression of downstream pathogenesis-related (*PR*) genes (Zhang et al. 2017; Chen et al. 2018). *Sr26* and *Sr61*, encoding two distinct NLR proteins, were sourced from *Thinopyrum ponticum* (Zhang et al. 2021). *Sr33* and *Sr35* were the first cloned ASR genes conferring resistance to stem rust race Ug99 (Periyannan et al. 2013; Saintenac et al. 2013). *Sr33* encodes a NLR protein orthologous to *Mla* genes in barley and rye (Krattinger and Keller 2016). The LRR domains of Sr35 directly recognise the AvrSr35 protein encoded by *AvrSr35*, one of the first *Avr* genes described in wheat rust, and activate downstream defence signalling (Salcedo et al. 2017). *Sr60* and *Sr62* encode TKP proteins (Chen et al. 2020; Yu et al. 2022). *Sr62* exhibited a high level of resistance against diverse stem rust races, whereas *Sr60* conferred partial resistance against a few *Pgt* isolates (Chen et al. 2020; Yu et al. 2022). Interestingly, the recently cloned *Sr43* encodes for an unusual protein kinase fused to two domains of unknown function (DUFs) in the C terminal (Yu et al. 2023). However, *Sr43* is sensitive to temperature where high-temperature conditions reduce the effectiveness of the gene.

## Genomic regions with pleiotropic effects on APR to rust diseases

### Cloned pleiotropic genes and catalogued loci

Two well-known cloned pleiotropic genes, *Lr34/Yr18/Sr57* and *Lr67/Yr46/Sr55*, confer partial and race-nonspecific resistance to multiple pathogens, including all three rust pathogens in wheat. *Lr34/Yr18/Sr57* is one of the most widely deployed gene regions in wheat breeding over the past century. Leaf tip necrosis is a visual characteristic of *Lr34/Yr18/Sr57* and used as a marker to assist selection in breeding programmes. *Lr34/Yr18/Sr57* encodes an ATP-binding cassette (ABC) transporter, involved in plasma membrane remodelling and shuttling various substrates, including a key substrate, phytohormone abscisic acid (ABA) (Krattinger et al. 2009; Deppe et al. 2018). The resistance allele *Lr34/Yr18/Sr57res* alters ABA-related processes and facilitates accumulation of multiple antifungal phenylproponoid metabolites (Krattinger et al. 2019; Rajagopalan et al. 2020). Another cloned gene conferring pleiotropic and quantitative resistance is *Lr67/Yr46/Sr55*, which encodes a hexose transporter, sugar transporter protein STP13 (Moore et al. 2015). *Lr67/Yr46/Sr55* might restrict the symport of H + /glucose molecules in infected leaves, causing disturbance of sugar balance in leaf cells and limitation of internal nutrients availability for pathogens. The introduction of *Lr34/Yr18/Sr57* or *Lr67/Yr46/Sr55* into other cereals, such as barley, demonstrated effective resistance against multiple pathogens, highlighting the utility of the genes and the highly conserved nature of resistance pathways in cereal crops (Risk et al. 2013; Sucher et al. 2017; Milne et al. 2019). These alleles provide difficult barriers for the pathogens to overcome due to low selection pressure for mutations, which supports longer-lasting multiple disease resistance, thus highly valuable for wheat breeding. Like *Lr34/Yr18/Sr57* and *Lr67/Yr46/Sr55*, other multi-resistance loci such as *Lr46/Yr29/Sr58* and *Sr2/Lr27/Yr30* have provided slow-rusting resistance for over 60 years. *Lr46/Yr29/Sr58* was the second formally named gene conferring slow-leaf-rusting resistance (Singh et al. 1998). Subsequently, *Lr46/Yr29/Sr58* was also reported to display pleiotropic resistance to stripe rust and stem rust, associated with leaf tip necrosis. Ongoing mapping and cloning studies are being conducted for *Lr46/Yr29/Sr58*. Cobo et al. (2019) fine-mapped *Lr46/Yr29/Sr58* to an interval of 332 kb on the distal region of chromosome arm 1BL, where 14 candidate genes were identified in the CS reference genome. Most of these were potentially related to plant defence, making it difficult to delineate the causal gene of *Lr46/Yr29/Sr58*. Another source of broad-spectrum APR is *Sr2/Lr27/Yr30*, which is conferred by a recessive resistance gene on chromosome 3BS (Spielmeyer et al. 2003). It was originally introduced from emmer wheat into common wheat. Different types of molecular markers have been well developed to assist the selection of *Sr2/Lr27/Yr30* in large breeding populations (Vishwakarma et al. 2019). The stacking of *Sr2/Lr27/Yr30* and *Lr34/Yr18/Sr57* resulted in significantly increased field APR to all the three wheat rusts (Randhawa et al. 2018). Recently, *Sr2/Lr27/Yr30* was coupled with its linked repulsion gene *Fhb1* for transfer into CIMMYT germplasm to improve the resistance of both Fusarium head blight and stem rust (He et al. 2020). Besides *Lr46/Yr29/Sr58* and *Sr2/Lr27/Yr30*, the slow-leaf-rusting gene *Lr68*, located on the long arm of chromosome 7B, was also found to confer some resistance to stem rust (Herrera-Foessel et al. 2012). Notably, wheat lines carrying both *Lr34/Yr18/Sr57* and *Lr68* display higher levels of resistance to leaf rust compared to those with a single gene (Randhawa et al. 2018). This highlights the potential of using *Lr68* in gene stacks to breed for multiple rust resistance.

### Genomic regions potentially harbouring loci with pleiotropic effects for multiple rust resistance

In addition to the four pleiotropic genes described above, we also identified 22 potential multi-rust resistance loci (i.e. associated with at least two wheat rusts) based on shared markers of QTL from different studies (Table S7; Fig. 1). These multi-disease QTL were distributed on chromosomes 1B (2), 2A (1), 2B (3), 2D (1), 3B (2), 3D (1), 4B (1), 5A (1), 5B (2), 5D (1), 6A (2), 6B (2), and 7B (3), respectively. Interestingly, one multi-disease QTL, MDQ7B.1 located on the long arm of chromosome 7B, appeared to affect all three rusts. The markers *Xcfa2040* and *Xwmc273* were associated with the locus. On the distal end of chromosome 7BL, several QTL were reported for rust resistance, including *Lr14a* conferring race-specific resistance, suggesting a hotspot of *R* genes. Further tests are required to rule out the existence of multiple independent loci in MDQ7B.1, for example using a large segregating population. Likewise, the pleiotropic effects should be validated for the other multi-disease QTL. Nevertheless, these insights are holding promise for the discovery of additional new pleiotropic rust resistance loci in future to support resistance breeding efforts.

## Exploiting the knowledge of rust resistance loci in wheat breeding

### Marker-assisted selection and genomic selection

**Fig. 2:**
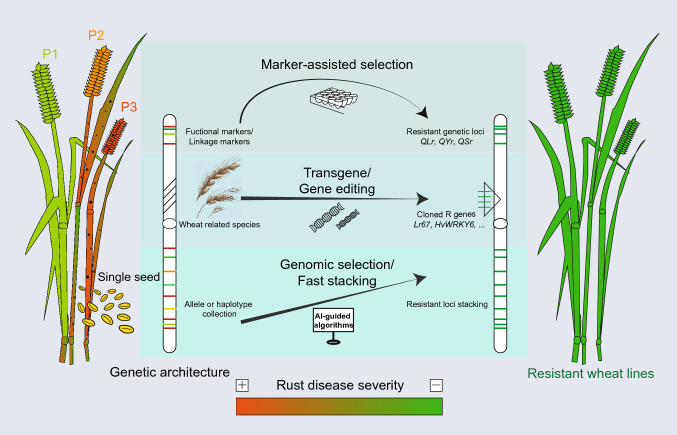
Schematic diagram of resistance breeding strategies by taking advantage of genetic architecture underlying rust diseases in wheat. Marker-assisted selection involves the transfer of resistance using closely linked or gene-specific markers when a transgene/gene-editing strategy introduces resistance through gene transfer or modifications. However, the fast approach is to stack multiple resistances through genomic selection with artificial intelligence (AI)-guided algorithms

Conventional breeding has achieved notable successes in enhancing wheat rust resistance through the accumulation of resistance alleles over cycles of selection. A range of resistance sources have also been used to conduct hybridization followed by intensive selection and field testing. For instance, exotic *R* genes have been successfully introduced into wheat lines, such as *Sr2/Lr27/Yr30* and *Sr25* derived from *T. turgidum* and *Th. obtusiflorum*, respectively (Pan 1940; Friebe et al. 1994). While successful, the timelines of conventional breeding tend to be lengthy, and there is the possible loss of target genes during breeding processes due to rapid evolution of pathogen races. Linked or diagnostic molecular markers provide supplementary tools to support traditional resistance breeding (Fig. 2). Diagnostic markers have been developed for most of the 35 cloned rust genes, facilitating their tracking and precise selection in breeding programmes. Diagnostic markers have been successfully implemented in both public and private breeding programmes. Diagnostic markers are also valuable for researchers to identify accessions with potentially novel resistance. For instance, the markers specific for APR genes *Lr34/Yr18/Sr57* and *Lr67/Yr46/Sr55* were used to screen for the presence of the corresponding resistance alleles in the Vavilov wheat diversity panel (Riaz et al. 2018). They can also provide insights into selection, as demonstrated by Zhou et al. (2022) where the authors screened 153 Chinese and CIMMYT cultivars using functional markers for stripe rust resistance genes. Furthermore, knowing the combination of resistance genes present in a population can help deepen our understanding of gene effects and their interactions.

Diagnostic markers for rust genes have been integrated to develop high-throughput and breeder-friendly markers in wheat, such as the KASP (Kompetitive Allele Specific PCR) marker platform and the GBTS (genotyping by target sequencing) liquid chip. A toolkit with over one hundred confirmed KASP markers, including SNPs that are associated with rust resistance loci, has been utilised to screen ~ 3,000 wheat lines in China (Rasheed et al. 2016; personal communication). A total of 60 disease resistance markers were also included in screening a wheat breeding panel based on GBTS technology, which is highly flexible and cost-effective (Guo et al. 2021). The 920 genetic loci summarised in this review offer a good entry point for establishment of a maker platform in wheat resistance breeding, and the 154 QRC could be prioritised (Table S2; Table S3). Taking full advantage of the latest CS reference genome, selected markers can be adjusted in the physical context, and sharable marker data allows the toolkit to be easily updated into the future.

For loci that are yet to be cloned, closely linked markers can be converted to user-friendly markers for breeding. However, validation should be performed to confirm associations between the markers and underlying genetic loci. For example, 41 of the 154 QRCs described in Fig. 1 could be targeted since they have been confirmed in separate bi-parental hybrid and diversity populations. One potential problem is that the linked marker(s) might not remain in the same LD block with the causal gene across generations due to recombination. Also, linkage drag between the resistance gene and other deleterious traits could be difficult to manage. For example, *Yr64* and *Yr65* were successfully stacked by using their linked markers, but undesirable traits were observed in multi-gene stack wheat lines (Cheng et al. 2014).

In contrast to marker-assisted selection (MAS), which targets a small number of markers, genomic selection (GS) uses the markers throughout the whole genome to predict genomic estimated breeding values (GEBVs) of candidate lines based on training populations and prediction models. The rate of genetic gain for rust resistance can be greatly improved using GS and does not require phenotyping of candidates (Xu et al. 2017). GS would be particularly useful for stacking a large number of loci with minor effect on rust resistance (Juliana et al. 2019). High genomic prediction accuracies (*r* values) of 0.76 and 0.73 were calculated in wheat leaf and stripe rust resistance, respectively, by using thousands of pre-selected significant and low-LD SNP markers from GWAS (Pang et al. 2021). When diagnostic markers were added into GS models or close relationships were found between validation and reference lines, prediction accuracy increased substantially for resistance against three wheat rusts (Daetwyler et al. 2014). Prediction models are critical in GS and the recent surge of nonlinear models, especially deep-learning algorithms, has facilitated the development of new prediction models that can integrate complex non-additive effects and additional data types beyond DNA markers. For example, a recently established deep neural network-based model (DNNGP) allows GS with multi-omics data and has been effectively used in wheat (Wang et al. 2022a). Provided large reference populations are used (i.e. large panels of genotyped lines with rust scores); high prediction accuracies for rust resistance are expected in breeding applications.

### Gene cloning and genetic engineering of rust resistance

In this review, we have summarised and provide new insights into the complex genetic architecture of rust resistance in wheat (Fig. 1). Most QTL were coarsely mapped using genetic populations generated by diverse parents with contrasting phenotypes. Importantly, potential resistance sources were identified for many QTL, which should contribute to the identification of causal genes and ultimately causal mutations underpinning these loci, especially QRCs containing loci reported in multiple independent studies. To exploit this information and accelerate the discovery of new casual rust resistance genes, a range of gene-cloning strategies can be employed including traditional map-based cloning, genomic-assembly assisted cloning, chromosome-sorting-based strategies, and *NLR* gene capture sequencing. In classical positional cloning, additional markers can be developed to saturate QTL regions and to genotype the large segregating or secondary populations constructed from crossing selected parents. Coupled with accurate recorded resistance phenotypes, the genetic interval of targeted QTL could be narrowed down to reduce the number of candidate genes. This is a resource-intensive and time-consuming process since high recombination rates through the evaluation of large segregating populations are necessary. Fortunately, the availability of wheat reference sequences (CS and 10 + wheat genomes) and gene transcription patterns (expVIP: http://wheat-expression.com/) aid in identifying candidate genes within QTL intervals (Ramírez-González et al. 2018; Walkowiak et al. 2020). Also, different online datasets are valuable resources. For example, WheatOmics 1.0 platform has combined several continuingly updating multi-omics resources to accelerate functional genomics studies in wheat (Ma et al. 2021). The rapid advent of genome sequencing with improved wheat genome assembly have greatly facilitated genomics-assisted cloning. A good example is the cloning of *Yr27*, where the whole genome of the donor source, Kariega, was subjected to long-read sequencing and a high-quality assembly was generated (Athiyannan et al. 2022a). Another gene-cloning strategy is to reduce complexity of the wheat genome in an unbiased and lossless manner. MutChromSeq and TACCA approaches have been developed based on isolation of specific chromosomes followed by a sequence comparison between the wild type and screened mutants to identify candidate genes (Sánchez-Martín et al. 2016; Thind et al. 2017). The main difficulty of such strategies is that flow sorting chromosomes requires very specific expertise. As a result of genome complexity reduction, RenSeq has been targeted for the largest *R* gene family, NLR protein encoding genes. This method has dramatically transformed the time required to clone a *R* gene, even for wheat with a large and complex genome. Deployment of MutRenSeq has enabled efficient isolation of several wheat rust *R* genes, including *Lr13*, *Yr5a*/*Yr5b*, *Yr7*, *Sr22*, *Sr26*, *Sr45*, and *Sr61* (Steuernagel et al. 2016; Marchal et al. 2018; Zhang et al. 2021; Hewitt et al. 2021). This approach could be used to isolate the causal genes underpinning major ASR QTL reported in this review. One limitation should be noted is that the design of capture baits is highly dependent on annotated genes and reference genome sequences. To this end, the recently released wheat pan-genome can help to capture diversity within *NLR*s (Walkowiak et al. 2020). Another limitation is that this approach is constrained to causal genes that belong to the *NLR* family only.

Gene editing using CRISPR/Cas9 systems offers potential to create new resistance alleles in elite material that has been identified in very diverse germplasm (Fig. 2). For instance, loss-of-function mutants can be generated for *Lr67sus* to prevent pathogen infection, and this has been demonstrated in different crops (Milne et al. 2019; Gupta et al. 2021). In most cases, *R* genes are dominant, and resistance is unlikely to be achieved by simply editing out the gene. The advancement of precision genome editing could provide the potential to create new alleles with desirable functions. A good example is the introduction of *Tamlo-R32*, a new allele of *mlo* created by precise editing. This edit conferred a high level of powdery mildew resistance in wheat without growth and yield penalties (Li et al. 2022). In addition, genome engineering by transforming individual or combined *R* genes is an effective way for increasing resistance in wheat (Fig. 2). A proof-of-concept study transferred five-*Sr*-gene cassettes into wheat to achieve high levels of field resistance to stem rust (Luo et al. 2021). Restrictions in many countries concerning the regulation of wheat classified as genetically modified (GM) are possibly the biggest barrier to widespread adoption of these tools (Caradus 2023).

### Fast stacking rust resistance loci by integrating computational simulation and speed breeding

A major challenge for wheat breeders is how to stack large numbers of resistance loci, such as the QRCs identified in this review or multi-pathogenic *R* genes, into elite varieties in the shortest possible time. This challenge becomes more difficult as the number of resistance alleles increase, which are now being discovered constantly and rapidly with genomic advances. For instance, if 12 parental lines each carry a different *R* gene (e.g. four resistance genes each for stripe, leaf and stem rusts) were to be stacked into an elite background, it would take 19 generations through crossing (Hafeez et al. 2021). The timeframe can be reduced if stacking begins by selecting the optimal set of parental lines that between them maximise favourable resistance alleles.

Extending the gene pyramiding concept of Hospital and Dekkers (1994), several strategies have been proposed to stack haplotypes with favourable effects on target traits. For example, Kemper et al. (2012) proposed a genetic algorithm, an artificial intelligence (AI)-based method, to stack desirable haplotypes into an “ultimate” genotype. Goiffon et al. (2017) extended this approach in their optimal population value (OPV) approach that predicts the theoretically optimal combination of haplotypes in a gamete and has increased genetic gain in a simulated wheat breeding programme using double haploids (Goiffon et al. 2017). Maximum haploid breeding value (EMBV) is another promising strategy predicting performance of the best gametes from a parental candidate (Müller et al. 2018). The approach of Kemper et al. (2012) is currently being applied to rapidly improve wheat yields (Villiers et al. 2024), but also holds promise for rapidly stacking disease resistance alleles (Fig. 2). The main function of the genetic algorithm is to identify a set of parents that together maximise the total value of best chromosome segments for rust resistance at each position along the entire genome. Subsequently, the AI-selected parents are used to optimally design a tailored introgression mating scheme. Employing digital twins created by computer simulations, different crossing scenarios are explored to determine the optimal strategy that most efficiently stacks alleles in the shortest possible time. The targeted mating and breeding design can be fast-tracked in a speed breeding facility that uses optimal light/temperature conditions to accelerate time to flowering and generation advance (Watson et al. 2018). Under optimal speed-breeding conditions, between four to six generations of spring wheat can be grown per year (Watson et al. 2018), greatly improving the genetic gain for accumulating multiple *R* genes. The methodology could also contribute to developing multi-disease resistant wheat lines beyond the rusts by addressing the trade-off or interactions between different diseases.

## Perspectives

In wheat, advances in genomics, phenotyping, and gene-cloning technologies will continue to accelerate the pace of genetics research on rust resistance, leading to continuous discovery of *R* genes or genetic loci. This review summarised current knowledge of the genetic basis of rust resistance, integrated mapped loci and cloned genes into a genome atlas, and identified a group of genomic regions that have been reported in multiple studies. The genetic landscape displayed in our review should enable pathologists and wheat breeders to easily interrogate the catalogued rust resistance QTL and genes. The sharable physical genome location enables fast comparisons between different studies. The integration and abundance of genetic resources are critical to combat epidemics of the three wheat rusts. The 26 potential pleiotropic loci identified in this review may warrant a greater focus for future research. The important genetic loci listed in our catalogue could also be used to help target gene-cloning or -editing studies. The ultimate goal of genetic dissection and gene discovery is to facilitate efficient molecular breeding for desirable traits in crops. Allelic variations of *R* genes need to be detected in diverse genetic resource collections, followed by the establishment of high-throughput and cost-effective MAS platforms. Furthermore, GS offers a powerful approach for breeders to predict breeding values for rust resistance and accelerate genetic gain for resistance in their breeding programmes. We anticipate that computational approaches for targeted and efficient pyramiding of superior haplotypes will be the next frontier for rust resistance breeding. When performing AI-based selections for fast-stacking resistance haplotypes, several factors should be taken into account. Firstly, as well as focusing on major *R* genes with large effects, minor genes could be targeted, particularly those with beneficial effects on multiple diseases could be given high priority in the genetic algorithm to maximise durable resistance, as stacking of four or five minor genes might achieve “near immunity” for rust disease resistance in wheat (Singh et al. 2014). Also, stacking *R* genes involved in diverse defence pathways could increase multi-rust resistance. For example, resistance to all three rusts can be enhanced significantly by combining *Sr2/Lr27/Yr30* and *Lr34*/*Yr18*/*Sr57* (Randhawa et al. 2018). A combination of *Lr34* and *Lr46* contributed to durable resistance in the wheat cultivar Carberry (Bokore et al. 2022). Strong background resistance resulting from an accumulation of minor genes is the key in CIMMYT resistance breeding efforts (Bhavani et al. 2019). Meanwhile, these minor genes can also enhance and prolong the efficiency of race-specific *R* genes, highlighting the value of stacking both minor and major *R* genes simultaneously (Brun et al. 2010). Secondly, epistatic and pleiotropic effects of selected *R* genes should be considered in haplotype stacking approaches. For example, *Pm64* and *Yr5* are linked in repulsion, making them difficult to deploy together (Zhang et al. 2019b). In such cases, the underlying causal gene with positive effect on resistance to one disease might reduce resistance levels to another. This phenomenon should be considered when improving multi-disease resistance to rusts and necrotrophic diseases, such as Septoria nodorum blotch (our unpublished data). A simple, but possibly effective idea, is to only stack the genetic regions favourable to multiple disease resistance. Another factor to be considered is resistance suppression, which is a common problem in gene introgression from lower to higher ploidy levels (Ma et al. 1995). This is probably attributed by suppressor or modifier genes present in the genetic background of wheat lines to be improved. As lower ploidy wild relatives are the important sources of *R* genes, resistance suppression occurs for all the three rust diseases. For example, *SuSr-D1* suppressing stem rust resistance encodes for a Med15 protein as a member of Mediator Complex (Hiebert et al. 2020). Functional suppression was found with reduced transcript level when introducing *Yr28* into synthetic hexaploid wheat lines (Athiyannan et al. 2022b). Finally, it is ideal to stack favourable resistance alleles or haplotypes without detrimental agronomic traits caused by linkage drag, perhaps through gene-editing. From a practical perspective, a trade-off or balance between *R* gene and plant growth must be achieved (Pokotylo et al. 2022). Maintaining overly high disease resistance by stacking too many *R* genes might consume considerable energy of host plants, thereby reducing yield to some extent. Thus, wheat cultivars with sufficient level of resistance and high yield could provide potentially more economic returns for farmers.

### Supplementary Information

Below is the link to the electronic supplementary material.

Supplementary file1 (PDF 573 kb)

Supplementary file2 (XLSX 188 kb)
