# Supplementary figures and images for: Genome-wide atlas of rust resistance loci in wheat
Source: Theor Appl Genet. 2024 Jul 9;137(8):179. doi: 10.1007/s00122-024-04689-8 (PMC11233289; doi:10.1007/s00122-024-04689-8)

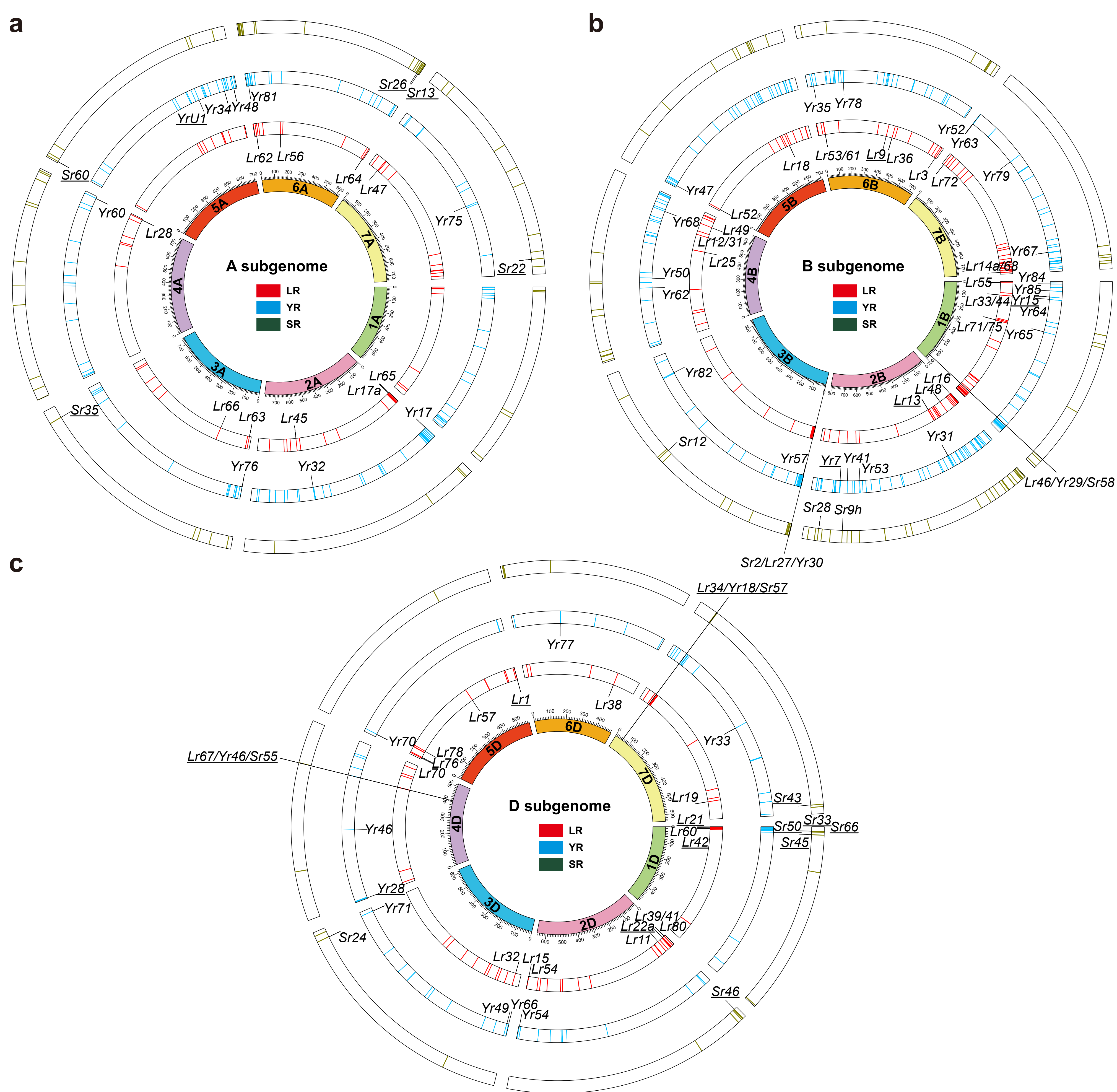

Supplement: Supplementary file 1 — Supplementary file1 (PDF 573 kb) [file 122_2024_4689_MOESM1_ESM.pdf]
